# Allergen-Specific Immunotherapy With Liposome Containing CpG-ODN in Murine Model of Asthma Relies on MyD88 Signaling in Dendritic Cells

**DOI:** 10.3389/fimmu.2020.00692

**Published:** 2020-04-23

**Authors:** Ricardo Wesley Alberca-Custodio, Lucas D. Faustino, Eliane Gomes, Fernanda Peixoto Barbosa Nunes, Mirian Krystel de Siqueira, Alexis Labrada, Rafael Ribeiro Almeida, Niels Olsen Saraiva Câmara, Denise Morais da Fonseca, Momtchilo Russo

**Affiliations:** ^1^Institute of Biomedical Sciences, Department of Immunology, University of São Paulo, São Paulo, Brazil; ^2^Center for Immunology and Inflammatory Diseases, Division of Rheumatology, Allergy and Immunology, Massachusetts General Hospital, Harvard Medical School, Boston, MA, United States; ^3^Department of Allergens, National Center of Bioproducts (BIOCEN), Havana, Cuba

**Keywords:** allergen, immunotherapy, dendritic cell, MyD88, asthma

## Abstract

Changing the immune responses to allergens is the cornerstone of allergen immunotherapy. Allergen-specific immunotherapy that consists of repeated administration of increasing doses of allergen extract is potentially curative. The major inconveniences of allergen-specific immunotherapy include failure to modify immune responses, long-term treatment leading to non-compliance and the potential for developing life-threating anaphylaxis. Here we investigated the effect of a novel liposomal formulation carrying low dose of allergen combined with CpG-ODN, a synthetic TLR9 agonist, on established allergic lung inflammation. We found that challenge with allergen (OVA) encapsulated in cationic liposome induced significantly less severe cutaneous anaphylactic reaction. Notably, short-term treatment (three doses) with a liposomal formulation containing co-encapsulated allergen plus CpG-ODN, but not allergen or CpG-ODN alone, reversed an established allergic lung inflammation and provided long-term protection. This liposomal formulation was also effective against allergens derived from *Blomia tropicalis* mite extract. The attenuation of allergic inflammation was not associated with increased numbers of Foxp3-positive or IL-10-producing regulatory T cells or with increased levels of IFN-gamma in the lungs. Instead, the anti-allergic effect of the liposomal formulation was dependent of the innate immune signal transduction generated in CD11c-positive putative dendritic cells expressing MyD88 molecule. Therefore, we highlight the pivotal role of dendritic cells in mediating the attenuation of established allergic lung inflammation following immunotherapy with a liposomal formulation containing allergen plus CpG-ODN.

## Introduction

Asthma is a complex chronic respiratory disorder characterized by episodes of cough and wheezing with increased mucus secretion that results in variable airflow limitation and airway hyper-responsiveness (1). Nowadays, it is becoming clear that asthma is a syndrome that encompasses various clinical phenotypes generally divided in “Th2 high” and “Th2 low” forms that correspond respectively to atopic or non-atopic asthma (2), with a wide variation in prevalence and severity (3). Patients with a high Th2 endotype show elevated levels of IL-4, IL-5, IL-9, and IL-13 in the airways (4, 5). These cytokines mediate allergic eosinophilic inflammation and isotype switching to IgE (6).

It is postulated that asthma can be either prevented or suppressed by intrinsic and/or extrinsic factors that collectively modify the immune responses to airborne allergens (7). Among extrinsic factors, the “hygiene hypothesis” gained special interest by postulating that early life infections are required for reduced predisposition to develop allergic diseases (8). However, the cellular and molecular mechanisms of the immunological switch against airborne allergens are still elusive. Different mechanisms such as maturation of the immune system, immune-deviation toward a Th1 profile, active suppression by different regulatory cells including Foxp3-expressing regulatory T (Treg) cells, IL-10-producing T or B regulatory cells, or anergy have been proposed (9). Whatever the mechanism, the clinical course of asthma suffers significant influence by life-style, nutrition, infections and microbial products (10). Indeed, bacteria and helminths in the digestive tract offer protection against asthma and allergies (11–14). Moreover, numerous reports have shown the inhibitory effects of bacterial components on allergic responses (15). For example, children that live in rural areas are exposed to higher concentrations of dust muramic acid, a constituent of peptidoglycan present in gram-negative and gram-positive bacteria, showed a lower prevalence of asthma and wheezing compared with children that live in urban areas (16). Experimentally, the hygiene hypothesis could be approached using microbial infections or microbial products that signal through toll-like receptors (TLR). Many bacterial components have previously been used in experimental asthma models aiming to improve the treatment effectiveness (17–20). We have previously shown that among TLR agonists studied, the TLR9 agonist CpG-oligodeoxynucleotides, hereafter denominated CpG, was the most effective in preventing allergic sensitization (21).

Changing the immune responses to allergens is the golden-standard of allergen-specific immunotherapy since its introduction in 1911 (22). Allergen specific immunotherapy consists of repeated and long-term applications of increasing doses of a particular allergen or group of allergens by subcutaneous or sublingual routes. However, there are numerous inconveniences of allergen-specific immunotherapy including failure to modify the immune system, long-term treatment that results in non-compliance, allergy exacerbation and the potential for developing systemic allergic reactions and life-threating anaphylaxis. Here, we sought to investigate the effect of a novel immunotherapy formulation in a murine model of asthma that consisted of short-term treatment (three doses) with low concentrations of allergen and CpG co-encapsulated in a cationic liposome composed of N-[1-(2,3-Dioleoyloxy) propyl] -NN, N, N-trimethylammonium-(DOTAP). The use of liposomal formulation to encapsulate allergen and CpG was chosen due to its ability to transport DNA, RNA, and other negatively-charged molecules into eukaryotic cells (23) and, therefore, with the potential to limit the contact of the allergen with anaphylactic antibodies and consequent anaphylaxis while enhancing CpG signaling through endosomal TLR9 (24).

## Materials and Methods

### Animals

Six-to-eight-week-old female 129S1, C57BL/6 mice (WT), MyD88-KO were originally purchased from Jackson Laboratories (Bar Harbor, ME). Mice expressing the recombinase Cre under the control of Itgax promoter (CD11c-Cre) (25) and *Myd88 fl/fl* mice (26) were bred together to generate CD11cMyD88^–/–^ (DC-MyD88^–/–^) and proper littermates CD11cMyD88^+/+^ (DC-MyD88^+/+^). Mice were kept in a specific pathogen-free breeding unit at the Institute of Biomedical Sciences of the University of São Paulo (ICB IV-USP). 10BiT that report Thy1.1 on the cell surface under the control of *Il10* promoter (27) were crossed with Foxp3^*gfp*^ reporter mice (28) to generate 10BiT.Foxp3^*gfp*^ dual reporter mice and were kept at specific-pathogen-free conditions at the animal facility of the Massachusetts General Hospital. All mice were kept in cages with a ventilated system, at a maximum of five animals per cage, with temperature-controlled rooms, food, and water *ad libitum*, in a 12 h light/dark cycle. Mice were treated according to animal welfare guidelines of ICB (Ethic Protocol 009/2015) under National Legislation-11.794 Law or under a study protocol approved by Massachusetts General Hospital Subcommittee on Research Animal Care.

### Experimental Protocol

Mice were sensitized subcutaneously (s.c.) to OVA (4 μg) or *Blomia tropicalis* (Bt) (4 μg) with alum adjuvant gel (1.6 mg) on days 0 and 7. Mice were intranasally challenged with OVA (10 μg) or Bt (10 μg) in 40 μL of PBS on days 14, 38 and 45. Mice were treated on days 17, 24, and 31 according to specifications in each experiment. The final concentrations or volume of each reagent in one dose of the liposomal formulation was: 4 μg of allergen (OVA or Bt), 10 μg of CpG and 70 μL of DOTAP. More specifically, for the preparation of the immunotherapy OVA + CpG/DOTAP was: OVA (4 μg) in a volume of 4 μL and CpG (10 μg) in a volume of 3 μL were slowly mixed, and 70 μL of DOTAP was added to the mixture and slowly mixed with the aid of the pipette for 1 min. The liposomal formulation was kept at room temperature for 15 min followed by the addition of 123 μL of PBS. Control mice consisted of non-manipulated animals. All procedures (sensitization, challenges, and treatment) were done under anesthesia with ketamine (50 mg/kg) and xylazine (20 mg/kg). On day 46, animals were euthanized with inhaled halothane and samples were collected.

### Reagents

CpG-ODN 2395 Class C, a TLR9 agonist, was purchased from Invivogen (San Diego, CA, United States). The allergens used were OVA (Sigma-Aldrich, United States) and *B. tropicalis* extract (BIOCEN, Cuba). The OVA was depleted of endotoxin (LPS) using four cycles of Triton X-114 extractions. The endotoxin level of purified OVA was below the limit of detection of Limulus assay lysate (less than 0.1 Endotoxin Units). DOTAP (DOTAP Liposomal Transfection Reagent) was purchased from Sigma–Aldrich (Sigma–Aldrich, St. Louis, MO, United States).

### Blood and Bronchoalveolar Lavage (BAL) Collections

Blood samples were collected by cardiac puncture, centrifuged, and serum was stored at –20°C. The BAL fluid was obtained after lung washing with 1 ml of cold PBS via the trachea. Total and differential cell counts of BAL fluids were determined by hemacytometer (Sigma–Aldrich, Sant Louis, United States) and cytospin (Thermo Fisher Scientific, Waltham, United States) preparation, stained with H&E (Instant-Prov, Newprov, Brazil), a stain based on Romanowsky formulation.

### ELISA for Cytokines

Cytokines (IL-5 and IFN-γ) levels in BAL were measured by sandwich kit enzyme-linked immunosorbent assay (ELISA) according to the manufacturer’s recommendation (BD Biosciences, United States). Values were expressed as pg/ml deduced from a standards curve of recombinant cytokines ran in parallel.

### Lung Harvest and Flow Cytometry

Lungs were digested with 0.52 U/ml Liberase TM (Roche) and 60 U/ml DNase I (Roche) in RPMI 1640 (Cellgro), at 37°C for 30 min. Lung leukocyte enrichment was performed by using a 30% Percoll gradient and the cell suspension obtained after erythrocyte lysis. Single cells were then incubated with anti-mouse CD16/32 (93, TruStain fcX, BioLegend) to block Fc receptors. Staining was performed with Fixable Viability Dye eF780 (eBioscience), to identify dead cells, and the following fluorochrome-conjugated anti-mouse monoclonal antibodies (mAbs): CD45-PerCP/Cy5.5 (30-F11), CD4-BV785 (GK1.5) (all from BioLegend), and CD3e-BUV395 (145-2C11) (from BD Biosciences). At least 1,000,000 events were recorded for each sample. Only viable and non-doublet cells were considered. Flow cytometric analysis was performed using a LSRFortessa X-20 flow cytometer (BD Biosciences) and FlowJo software (Tree Star).

### Active Cutaneous Anaphylaxis (ACA) Assay

Mice were sensitized as described above and challenged intranasally on day 14. On day 16, the dorsal region was shaved with a trimmer ER389 (Panasonic, Japan) and on day 17, the animals were anesthetized with ketamine and xylazine and received an intradermal injection of OVA (10 ug) or PBS followed by an intravenous injection of 100 μl of Evans Blue dye (1 mg/mL). Thirty minutes later, the animals were euthanized and the skin of the dorsal region was removed for photographic registration. Skin spots were weighted and dye extraction performed with formamide (8 mL/mg of dry weight) for 72 h (29) and quantified by measuring dye absorbance at 620 nm. Results are expressed as μg/mL determined by a standard curve with known concentrations of Evans blue dye.

### Lung Histopathology

After the BAL collection, 10 mL of cold PBS was perfused through the right ventricle of the heart, the lungs were fixed in 10% PBS-formalin for 24 h and then in 70% ethanol until embedding in paraffin. Sections of five-micrometer were stained with hematoxylin/eosin for determinations of lung inflammation or periodic acid-Schiff for mucus quantification. Lung inflammation score was performed by measurement of the peribronchial cellular infiltrates divided by the girth of the bronchial basal membrane. The mucus score was calculated as the percentage (%) of mucus positive area divided by the girth of the bronchial basal membrane.

### ELISA for Antibody Determinations

Mice were euthanized with overdose of anesthesia and blood samples were collected by cardiac puncture, centrifuged and serum was stored at -20°C. Serum antibodies were determined by ELISA as described previously (30).

### Statistical Analysis

Statistical analyses were performed using the software GraphPad Prism (V.5; GraphPad Software, United States). One-way ANOVA followed by Tukey post-test was performed, as appropriate. Differences were considered statistically significant when *p*-value ≤ 0.05. Data represent the mean ± SE.

## Results

### Allergen (OVA) Encapsulation in a Cationic Liposome Attenuates Active Cutaneous Anaphylaxis

**FIGURE 1:**
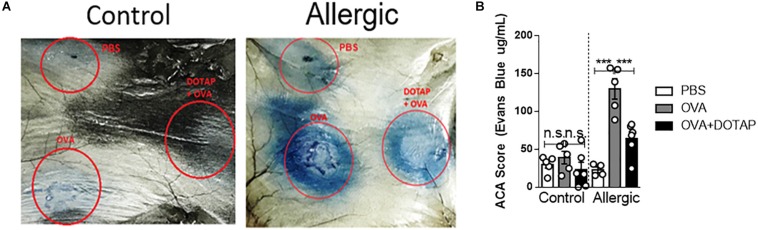
Active cutaneous anaphylaxis with free or encapsulated allergen. C57BL/6 wild-type mice were sensitized with OVA/Alum on days 0 and 7 and challenged with OVA on day 14 to establish airway inflammation. Control group consisted of non-manipulated naive mice. On day 17 (Control and Allergic) groups received an intradermal injection of PBS or OVA alone or OVA encapsulated in DOTAP liposome in the dorsal region followed by i.v. injection of Evans Blue dye. **(A)** Representative pictures of active cutaneous anaphylaxis (ACA) in the dorsal skin 30 min after Evans Blue dye injection in Control or Allergic groups; **(B)** ACA scores were determined in Control or Allergic groups after Evans Blue dye extraction with formamide as described in M&M. Each symbol represents one mouse. Values represent the mean ± SEM and are representative of three independent experiments. One-way ANOVA: ***p < 0.001; n.s. non-significant.

A major problem of allergen-specific immunotherapy is the development of life-threating anaphylaxis during treatment (31, 32). To circumvent this side effect, we postulated that encapsulation of the allergen (OVA) in a cationic liposome (DOTAP) would reduce the risks of anaphylaxis by preventing the allergen from the contact with allergen-specific IgE (33). To test this, we performed an ACA assay in C57BL/6 mice sensitized with OVA/Alum s.c. on days 0 and 7 and challenged with i. n OVA on day 14 (Allergic). ACA was induced with intradermal injection (i.d.) of OVA at day 17 in the dorsal region followed by i. v Evans Blue dye injection. Dye extravasation in the dorsal skin was measured 30 min later. As shown in Figure 1, challenge with encapsulated OVA (DOTAP + OVA group) in allergic mice, resulted in a significant decrease of dye extravasation when compared with non-encapsulated OVA group (Figures 1A,B). As expected, we did not observe any anaphylactic cutaneous reactions in non-sensitized (Control) group (Figures 1A,B). Thus, encapsulated OVA in cationic liposome trigger less severe allergen-induced ACA than non-encapsulated OVA.

### Treatment With Liposomal Formulation Containing Allergen Plus CpG Reverses Established Asthma

**FIGURE 2:**
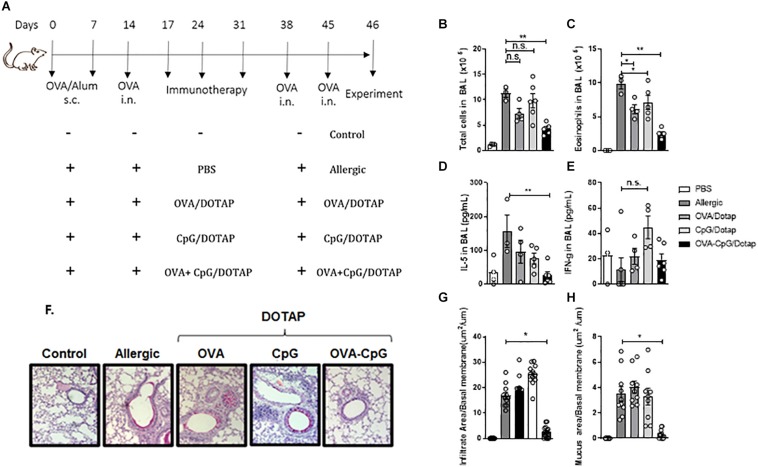
Liposomal formulation containing allergen plus CpG attenuates airway inflammation. C57BL/6 WT mice were sensitized s.c. with OVA/Alum on days 0 and 7 and challenged with i.n. OVA on days 14 to establish airway inflammation. Mice were treated subcutaneously with PBS (Allergic); or with encapsulated OVA in DOTAP (OVA/DOTAP); or with encapsulated CpG in DOTAP (CpG/DOTAP); or with OVA and CpG co-encapsulated in DOTAP (OVA + CpG/DOTAP) on days 17, 24 and 31 and challenged with i.n. OVA on days 38 and 45. Experiments were performed on day 46. Control group consisted of non-manipulated naive animals. **(A)** Schematic protocol of immunotherapy. **(B)** Total number of cells and **(C)** eosinophil counts; **(D)** IL-5 and **(E)** IFN-gamma levels in BAL. **(F)** Representative microphotographs of lung sections stained with periodic acid-Schiff, **(G)** lung inflammation score and **(H)** lung mucus score. Each symbol represents one mouse. Values represent the mean ± SEM and are representative of two independent experiments. One-way ANOVA: *p < 0.05; **p < 0.01; n.s. non-significant.

Since CpG signals through endosomal TLR9 (34), its activity could be increased after endocytosis of the liposome containing CpG. Therefore, we investigated whether treatment with a liposomal formulation containing the allergen plus CpG could alter the immune response of mice with established asthma following OVA challenge. For this, we compared mice with established allergic airway inflammation treated subcutaneously on days 17, 24, and 31 with PBS (Allergic group) with mice treated with OVA in liposome alone (OVA/DOTAP), or with CpG in liposome alone (CpG/DOTAP), or with OVA plus CpG in liposome (OVA + CpG/DOTAP) and challenged with OVA on days 38 and 45 as despicted in Figure 2A. Control group consisted of non-manipulated naive animals. Experiments were performed 24 h after the last OVA challenge on day 46. Allergic group had increased number of total cells and eosinophils in the BAL after OVA challenges when compared with Control group (Figures 2B,C). All groups treated with liposome in combination with OVA (OVA/DOTAP), CpG (CpG/DOTAP), or OVA + CpG (OVA + CpG/DOTAP) showed a significant reduction in the number of eosinophils in the BAL when compared with the Allergic group (Figure 2C). However, only the OVA + CpG/DOTAP group had a dramatic inhibition in the number of total cells and eosinophils, and a robust reduction in the levels of IL-5, but not IFN-γ levels in the BAL compared with Allergic group (Figures 2B–E). No differences were observed in the numbers of neutrophils or in the levels of IL-12 in BAL (data not shown). Notably, histopathological analysis of lung sections stained for H&E or periodic acid-Schiff (PAS) revealed that only animals treated with the liposomal formulation containing OVA plus CpG had a significant reduction in peribronchovascular inflammatory cell infiltrates and mucus formation, respectively (Figures 2F–H). We also determined the effect of liposomal formulation containing OVA and CpG on antibody production. We found that the serum levels of total IgE and OVA-specific IgE and IgG1 or IgG2c were increased in Allergic group when compared to Control group (Supplementary Figure S1). Treatment with the liposomal formulation reduced the total levels of IgE when compared with non-treated Allergic group (Supplementary Figure S1A). OVA-specific IgE and IgG1 were similar in OVA + CpG/DOTAP and Allergic group (Supplementary Figures S1B,C) while OVA-specific IgG2c antibodies reached higher levels in OVA + CpG/DOTAP group when compared to the Allergic group (Supplementary Figure S1D). Altogether, our results indicate that treatment with allergen plus CpG encapsulated in cationic liposome attenuates all parameters of Th2-mediated allergic lung inflammation and total IgE production, increases IgG2c production and does not affect the levels of OVA-specific IgE and IgG1 isotypes.

### Treatment With Liposomal Formulation Is Long-Lasting, Not Restricted to C57BL/6 Mouse Strain and Is Extended to Allergens Derived From House Dust Mite

**FIGURE 3:**
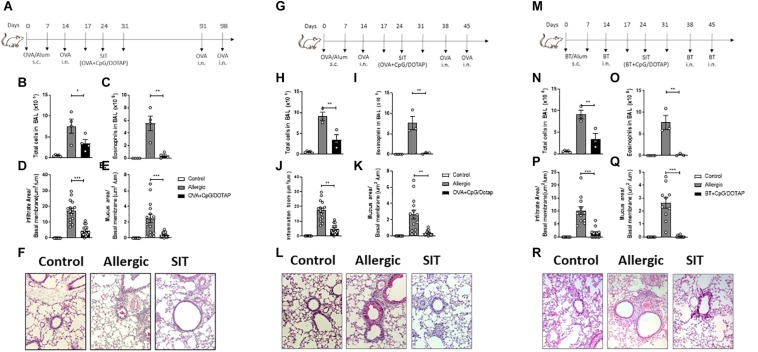
Treatment with liposomal formulation is long-lasting, not restricted to C57BL/mice and is extended to allergens derived from *Blomia tropicalis*. C57BL/6 WT mice were sensitized s.c. with OVA/Alum on days 0 and 7 and challenged with i.n. OVA on days 14 to establish airway inflammation. Mice were treated subcutaneously with PBS (Allergic) or with co-encapsulated OVA and CpG in DOTAP (OVA + CpG/DOTAP) on days 17, 24 and 31. **(A)** Schematic representation of the long-lasting allergen-specific immunotherapy protocol. Mice were challenged with i.n. OVA on days 91 and 98 and experiments were performed on day 99. Control group consisted of non-manipulated naive animals. **(B)** Total number of cells and **(C)** eosinophil counts in the BAL; **(D)** Lung inflammation score and **(E)** lung mucus score. **(F)** Representative microphotographs of lung sections stained with periodic acid-Schiff. **(G)** Schematic representation of allergen-specific immunotherapy protocol. 129S1 mice were sensitized with OVA/Alum s.c. on days 0 and 7 and challenged with OVA i.n. on days 14 to establish airway inflammation. Mice were treated subcutaneously with PBS (Allergic) or with co-encapsulated OVA and CpG in DOTAP (OVA + CpG/DOTAP) on days 17, 24 and 31 challenged with i.n. OVA on days 38 and 45. Experiments performed on day 46. **(H)** Total number of cells and **(I)** eosinophil counts in the BAL. **(J)** Lung inflammation score and **(K)** lung mucus score. **(L)** Representative microphotographs of lung sections stained with periodic acid-Schiff. **(M)** Schematic representation of allergen-specific immunotherapy protocol. C57Bl/6 wild-type (WT) mice were sensitized s.c. with *Blomia tropicalis* (Bt)/Alum on days 0 and 7 and challenged with i.n. Bt on day 14 to establish airway inflammation. Mice were treated subcutaneously with PBS (Allergic) or treated with (Bt) and CpG encapsulated in DOTAP (Bt + CpG/DOTAP) on days 17, 24, and 31 and challenged with i.n. Bt on days 38 and 45. Experiments were performed on day 46. **(N)** Total number cells and **(O)** eosinophil counts in the BAL. **(P)** Lung inflammation score and **(Q)** lung mucus score; **(R)** Representative microphotographs of lung sections stained with periodic acid-Schiff. Each symbol represents one mouse. Values represent the mean ± SEM and are representative of two independent experiments. One-way ANOVA: *p < 0.05; **p < 0.01; ***p < 0.001; n.s. non-significant.

Allergen-specific immunotherapy aims to provide sustained inhibition of the disease symptoms (35). Given that T cell memory is long-lasting in the OVA-induced allergic respiratory model (36) and that allergen-specific immunotherapy usually require many weeks or years of treatment (37, 38) we then next investigated whether our short-term (three-dose) treatment provides a long-lasting effect. For this, after the treatment, the mice were rested for 60 days and then challenged twice with i.n. OVA at days 91 and 98 as depicted in Figure 3A. We found that while the total number of cells and eosinophils were increased in BAL of allergic mice after OVA challenges compared to Control group, the treatment with liposomal formulation containing allergen and CpG significantly reduced the total cell and eosinophil counts in BAL when compared to Allergic group (Figures 3B,C). Histological analysis confirmed that the treatment was also effective in reducing lung allergic inflammation and mucus formation (Figures 3D–F). It is important to note that allergen-specific immunotherapy require several weeks or years of treatment (37, 38) while our treatment with liposomal formulation containing allergen and CpG was effective using a short-term treatment and low dose of allergen. In order to determine whether the efficacy of our short-term protocol treatment could be effective in other mouse strain, we tested our liposomal formulation in the 129S1 mice (Figure 3G), a mouse strain that is widely used in the production of targeted mutations (39). We found that the treatment with the liposomal formulation significantly inhibited all analyzed parameters of allergic inflammation such as airway total cell count and eosinophilia in BAL, peribronchial inflammatory infiltrates and mucus formation (Figures 3H–L), indicating that the treatment was also effective in 129S1 mouse strains. Finally, we aimed to determine whether liposomal formulation with specific allergen plus CpG could also be effective using allergens extracted from *B. tropicalis* (Bt) mite, a clinical relevant respiratory allergen. For this, we used a model recently described by our group that consists of subcutaneous sensitization with Bt mite extract adsorbed to alum adjuvant and a challenge with Bt i.n. to establish airway inflammation (40), followed by treatment with Bt + CpG liposomal formulation and challenges with Bt i.n. Allergic inflammation was determined 24 h after the last Bt i.n. challenge as depicted in Figure 3M. As in the OVA model, the treatment with the Bt + CpG liposomal formulation (Bt + CpG/DOTAP group) significantly inhibited the total number of cells and eosinophils in the BAL, as well as diminished the peribronchial inflammatory cell infiltrates and mucus formation in the lungs when compared with Allergic group (Figures 3N–R). We conclude that treatment with co-encapsulated allergen with CpG is effective in attenuating established asthma induced in a different mouse strains as well as induced by different allergens.

### Attenuation of Established Asthma by Liposomal Formulation Is Not Associated With Increased Number of Lung Regulatory T Cells

**FIGURE 4:**
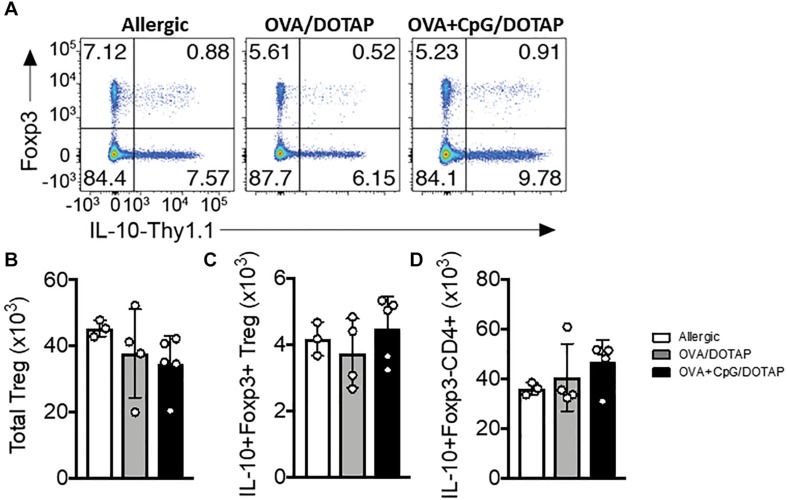
Regulatory T cells in the lung after allergen-specific immunotherapy. 10BiT.Foxp3^*gfp*^ mice were sensitized with OVA/Alum s.c. on days 0 and 7 and challenged with OVA i.n. on day 14 to establish airway inflammation. Mice were treated subcutaneously with PBS (Allergic) or with encapsulated OVA in DOTAP (OVA/DOTAP), or with co-encapsulated OVA and CpG in DOTAP (OVA + CpG/DOTAP) on days 17, 24 and 31 and challenged with i.n. OVA on days 38 and 45. Experiments were performed on day 46. Control group consisted of non-manipulated naive animals. **(A)** Flow cytometric analysis showing expression of Foxp3 and Thy1.1 (IL-10) in lung CD4^+^ T cells. **(B)** Total number of Foxp3^+^ Treg cells, **(C)** IL-10^+^Foxp3^+^ Treg cells, and **(D)** IL-10^+^Foxp3^-^CD4^+^ T (Tr1) cells. Each symbol represents one mouse. Values represent the mean ± SEM and are representative of one independent experiment.

Previous studies indicate that reduced activity of regulatory T cells, such as IL-10-producing Tr1 cells or Foxp3^+^ Treg cells is associated with allergic disease (41, 42) and that allergen-specific immunotherapy might increase their activity (43). To investigate the Foxp3^+^ and IL-10-producing regulatory T cell response *in vivo*, we crossed mice that express the Thy1.1 reporter under the control of *Il10* (10BiT) with mice that express the green fluorescent protein (GFP) under the control of *Foxp3* (Foxp3^*gfp*^) to generate 10BiT.Foxp3^*gfp*^ mice (27). These “dual-reporter” mice enable simultaneous detection of *Il10* and *Foxp3* expression in individual cells. Interestingly, we did not observe any difference in the percentage and number of Foxp3^+^ Treg cells, or in putative IL-10-producing Foxp3^+^ Treg or Foxp3^–^ Tr1 cells in the lung of allergic mice treated with the liposomal formulation OVA/DOTAP or OVA + CpG/DOTAP when compared with allergic mice treated with PBS (Allergic group) (Figures 4A–D). Thus, the inhibition of allergic lung inflammation by liposomal formulation in our OVA-model was not associated with an expansion of Foxp3^+^ or IL-10-producing regulatory T cells in the lung or a cell-based increase in IL-10 production.

### MyD88 Adaptor Molecule Is Essential for the Immunotherapeutic Effect of Liposomal Formulation

**FIGURE 5:**
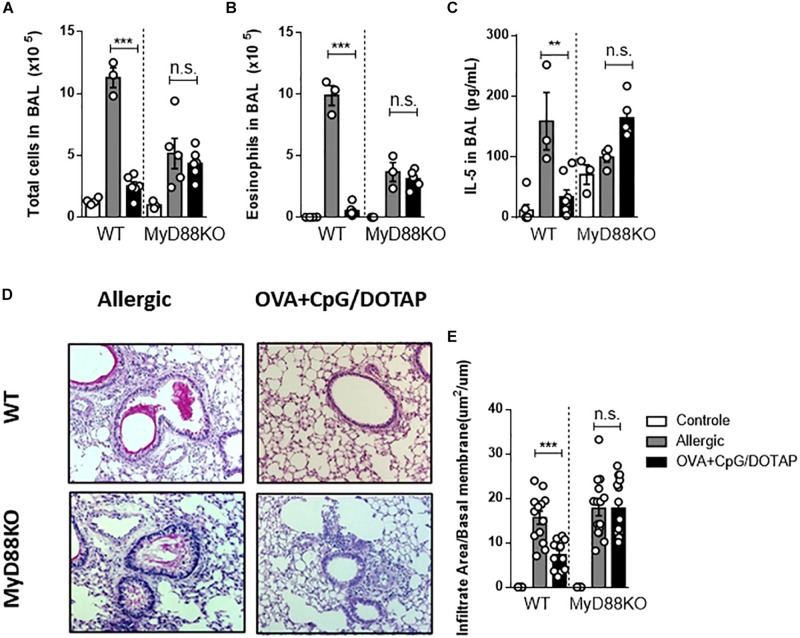
Involvement of MyD88 adaptor molecule in allergen-specific immunotherapy. C57BL/6 wild-type (WT) or MyD88-deficient (MyD88KO) mice were sensitized s.c. with OVA/Alum on days 0 and 7 and challenged with i.n. OVA on days 14 to establish airway inflammation. Mice were treated subcutaneously with PBS (Allergic) or with co-encapsulated OVA and CpG in DOTAP (OVA + CpG/DOTAP) on days 17, 24 and 31 and challenged with i.n. OVA on days 38 and 45. Experiments were performed on day 46. Control group consisted of non-manipulated naive animals. **(A)** Total number of cells and **(B)** eosinophil counts in BAL. **(C)** IL-5 levels in BAL. **(D)** Representative microphotographs of lung sections stained with periodic acid-Schiff. **(E)** Inflammation score. Each symbol represents one mouse. Values represent the mean ± SEM and are representative of two independent experiments. One-way ANOVA: *p < 0.05; **p < 0.01; ***p < 0.001; n.s., non-significant.

CpG signals through endosomal TLR9 via the MyD88 pathway (44), although it has been reported that high doses of CpG could signal through TRIF pathway (45). We have previously demonstrated that CpG has a prophylactic effect in allergic asthma that is dependent of MyD88 signaling (21). Here we sought to determine whether MyD88 signaling is also crucial for the therapeutic effect our liposomal formulation containing OVA + CpG. Using MyD88-deficient mice (MyD88KO) and C57BL/6 wild-type (WT) mice we found that treatment with liposomal formulation reduced the number of total cells, eosinophils, and the levels of IL-5 in the BAL in WT but not in MyD88KO mice when compared with the respective Allergic groups (Figures 5A–C). Further, histopathological analysis of lung sections confirmed that WT but not MyD88KO treated with OVA + CpG/DOTAP showed attenuated lung infiltration compared with the Allergic group (Figures 5D–E). These results show that MyD88 signaling is required to the attenuation of the established allergic lung inflammation induced by treatment with the liposomal formulation containing allergen and CpG.

### Dendritic Cells Expressing MyD88 Molecule Are Necessary and Sufficient for Reversal of Established Asthma by Liposomal Formulation

**FIGURE 6:**
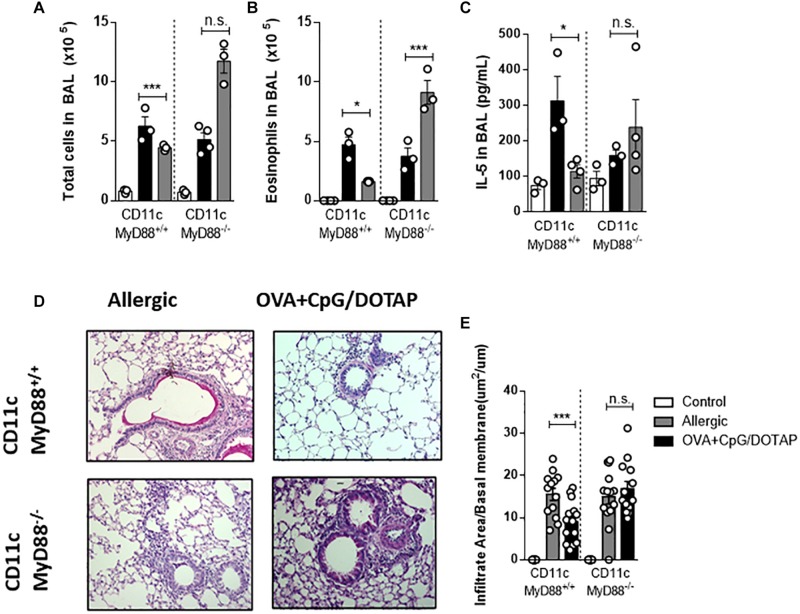
Dendritic cells expressing MyD88 molecule are fundamental for allergen-specific immunotherapy. Mice lacking *Myd88* expression on CD11c-positive dendritic cells (DC-MyD88^-^^/^^-^ and littermate controls (DC-MyD88^+/+^) were sensitized with OVA/Alum s.c. on days 0 and 7 and challenged with i. n OVA on day 14 to establish airway inflammation. Mice were also treated subcutaneously with PBS (Allergic) or treated with OVA and CpG encapsulated in DOTAP (OVA + CpG/DOTAP) on days 17, 24, and 31 and challenged with i.n. OVA on days 38 and 45. Experiments were performed on day 46. Control group consisted of non-manipulated naive animals. **(A)** Total number of cells and **(B)** eosinophil counts in BAL. **(C)** IL-5 levels of in BAL. **(D)** Representative microphotographs of lung sections stained with periodic acid-Schiff. **(E)** Lung inflammation score. Each symbol represents one mouse. Values represent the mean ± SEM and are representative of two independent experiments. One-way ANOVA: *p < 0.05; ***p < 0.001; n.s., non-significant.

Recently we showed that dendritic cells (DCs) expressing MyD88 molecule are crucial for CpG-induced inhibition of IgE production (30). Therefore, we next focused on the role of *Myd88* expression on dendritic cells known to respond to CpG signaling and are involved in antigen presentation (30, 46, 47). For this, we used mice with specific deletion of *Myd88* gene on cells expressing CD11c integrin (DCs-MyD88^–/–^) and their littermates’ controls (DCs-MyD88^+/+^). We found that DC-MyD88^+/+^ and DC-MyD88^–/–^ mice do develop allergic lung inflammation. Importantly, treatment with liposomal formulation containing co-encapsulated OVA and CpG reduced the number of total cells and, eosinophils in BAL as well as IL-5 production in DCs-MyD88^ + /^^+^, but not in DCs-MyD88^–/–^ mice when compared with the respective Allergic groups (Figures 6A–C). DC-MyD88^+/+^ mice also showed reduced lung inflammation as revealed by lung histopathology analysis compared with DC-MyD88^–/–^ mice treated with liposomal formulation (Figures 6D–E). Thus, our data indicates that the therapeutic effect of liposomal formulation in established asthma requires CpG signaling through MyD88 molecule expressed on CD11c-positive putative DCs.

## Discussion

Currently, pharmacological and immunological interventions are used for asthma control. Pharmacological treatment aims to control airway inflammation by the use of oral or inhaled corticosteroids and long-acting bronchodilators and as such the treatment is essentially symptomatic. Immunological treatment with monoclonal antibodies against anaphylactic IgE or against type 2 cytokines such as IL-5 and IL-4/IL-13 is another way to control asthma symptoms. In contrast, allergen-specific immunotherapy, a type of therapy that has been used in humans for more than a century (22, 48), has the potential of changing definitively the immune responses to specific allergens and has been indicated for non-responsive patient to pharmacological treatment (22, 49). Conventional allergen-specific immunotherapy consists of administration of repeated and increasing doses of the sensitizing allergen for long periods of time (35). In the present study, we introduced some variables to conventional allergen-specific immunotherapy, such as: (1) Instead of free-allergen we used encapsulated allergen in cationic liposome in order to avoid interaction with anaphylactic antibodies; (2) We added CpG to cationic liposome containing allergen to target directly endosomal TLR9; and (3) We used short-term (three doses) immunotherapy treatment and a low dose of allergen. We first found that the encapsulation of OVA alone (without CpG) in liposome was indeed effective in attenuating ACA as well as systemic anaphylaxis (data not shown). However, it is notewhorty that immunotherapy with encapsulated OVA alone, without CpG, was ineffective in reversing established allergic lung inflammation. Currently many variations of immunotherapy are being evaluated such as peptide immunotherapy (50), sublingual immunotherapy (51), intranasal (52, 53), oral (54) and subcutaneous (55). The gold standard for immunotherapy route is the subcutaneous route due to its efficacy (56), but sublingual treatment is on the rise. Our finding with encapsulated OVA is of clinical relevance since one of the major drawbacks of allergen-specific treatment is the development of life-threating anaphylaxis (57). Although treatment of allergic mice with encapsulated allergen alone was effective in attenuating cutaneous anaphylaxis of allergic mice, this treatment was ineffective, in reversing established allergic lung inflammation. Also, treatment with encapsulated CpG was also ineffective in reversing airway eosinophilic inflammation, a finding that is in line with a previous report (20). However, in contrast to our findings, allergen-free immunotherapy has been evaluated previously in a model of house dust mite asthma, using CpG and unrelated proteins from *Mycobacteria tuberculosis* cultures. It was found that the experimental respiratory allergy was down modulated when mice were treated concomitantly with CpG and culture proteins from *M. tuberculosis*. The inhibition of respiratory allergy was IFNγ-dependent indicating that immune responses to proteins of *M. tuberculosis* inhibited Derp1-induced allergic responses by a bystander mechanism (58). In addition, it was shown that encapsulation of CpG could improve its stimulatory effect signaling through endosomal TLR9 (26, 59–61) without increasing the toxicity of the compound (62–64). Notably, in our model, treatment with a liposomal formulation containing both OVA and CpG, but not CpG alone, reversed established asthma, attenuating allergic lung inflammation and decreasing type 2 cytokine production in BAL without increasing airway IFN-γ production. We showed previously that IFN-γ was not involved in CpG-induced inhibition of allergic sensitization (21, 30, 40). In contrast, some reports demonstrate that CpG (65) or OVA plus CpG or co-encapsulation of OVA and CpG (66) resulted in increased IFN-γ production. These differences might be related to different protocols used, the compartment of the body where IFN-γ was detected and whether the measurements were performed *in vitro* or *ex vivo*. More specifically, our results are in line with the work of Jain et al. (67) that showed that mucosal (nasal) administration with CpG and OVA reversed established allergic inflammation, reducing type 2 cytokines production in BAL without changing IFN-γ levels in BAL. In line with Jain et al. (67), we also found that our liposomal formulation was effective in attenuating established asthma when administered by the intranasal route without increasing IFN-γ (data not shown). Besides lung inflammation, allergen-specific immunotherapy aims to module also anaphylactic antibody productions. We found that our treatment with liposomal formulation containing OVA and CpG reduced the serum levels of total IgE but not the production OVA-specific IgE or IgG1 while it increased the production of OVA-specific IgG2c antibodies when compared with allergic group (Supplementary Figure S1). How these findings are related to the attenuation of allergic inflammation remain elusive. It is anticipated that either adaptive memory T cells (immune deviation) or regulatory T cells might participate in this process. Because we did not find evidence for IFN-γ-mediated immune deviation, we focused on the participation of regulatory T cells and IL-10 in the inhibition of allergic inflammation. Therefore, we investigated whether inhibition of established allergic lung inflammation could be associated with increased number of IL-10-producing regulatory Tr1 cells or with Foxp3-positive Tregs cells in the airways. Our results with IL-10 and Foxp3 double reporter mice clearly indicated that reversal of allergic inflammation by treatment with co-encapsulated OVA and CpG in cationic liposome was not associated with an increase of IL-10-producing putative Tr1 cells or with Foxp3-expressing putative Tregs after last OVA challenge. However, we cannot exclude de participation of regulatory T cell in other body compartments outside of the lung. Since the exact mechanism by which established adaptive Th2-mediated allergic responses were affected remained elusive, we focused on the role of MyD88 adaptor molecule in this process. CpG activates different innate cell types mainly signaling through MyD88 pathway via TLR9 (68), although it was reported that at high dose, CpG signaling through the TRIF pathway inhibited allergic bronchopulmonary aspergillosis (45). We first verified that MyD88 molecule was essential for CpG-mediated attenuation of the allergic reaction, ruling out the participation of TRIF pathway in this process. Since CpG activates different innate cell types (47, 69, 70) we next tested the participation of MyD88 molecule on CD11c-positive dendritic cells. For this, we used mouse engineered via Cre-lox technology with specific ablation of the mouse *Myd88* gene in CD11c-positive putative dendritic cells (25). As a control we used littermates mice with MyD88-expressing CD11c-positive cells. We found that DCs expressing MyD88 molecule were necessary and sufficient for CpG-induced attenuation of established asthma. Our results with DC-MyD88^–/–^ mice reinforce the notion that DCs expressing MyD88 molecule are key target cells for CpG-mediated immunomodulation (21, 26, 30, 71). Allergen-specific immunotherapy require long-term treatment and adherence to the treatment (72) to achieve protection against allergic reactions and symptoms (73). Usually, successful treatment is long lasting (38), but there are some conflicting reports as to whether the protective effect of immunotherapy persist after discontinuation (74, 75). It appears that the optimum duration for long-term modulation of allergic symptoms in humans by allergen-specific immunotherapy is approximately three years (76), while in mice allergen-specific immunotherapy appears to require at least 8-weeks (77). Here we showed that short-term treatment with low dose of allergens and CpG efficiently reduced key features of allergic asthma. In addition, we also evaluated whether this type of immunotherapy could be applied to a relevant respiratory allergens obtained from *B. tropicalis* mite extract, one of the most prevalent mites in tropical countries (78) and found that the treatment with co-encapsulated Bt allergens and CpG reversed the established allergic lung responses. These results demonstrate the potential applications of our formulation to different allergens and highlight the importance of correct diagnosis for developing a specific allergen treatment (79). Since allergic T cell memory is long-lasting (80), we investigated whether our immunotherapy protocol had a long lasting effect by performing experiments 2 months after the end of the immunotherapy. We found that our allergen-specific immunotherapy has a long lasting modulation on OVA-induced allergic responses. Collectively, our work highlights an improved short-term immunotherapy protocol using co-encapsulated allergens and CpG that has the advantage to protect against anaphylaxis during allergen-specific immunotherapy treatment, which uses low doses of allergens, is effective against different allergens and is long lasting. Our results suggest that this type of immunotherapy might be of potential use to treat eosinophilic (type 2 high) asthma endotype (2, 81) and indicate the pivotal role of dendritic cell expressing *Myd88* in this process.

## Data Availability Statement

All datasets generated for this study are included in the article/Supplementary Material.

## Ethics Statement

Mice were treated according to animal welfare guidelines of ICB (Ethic Protocol 009/2015) under National Legislation-11.794 Law or under a study protocol approved by the Massachusetts General Hospital Subcommittee on Research Animal Care.

## Author Contributions

RA-C designed the study, performed the experiments, analyzed the results, and wrote the manuscript. LF performed the experiments with IL-10 reporter mice and contributed to writing. MS, DF, FN, and EG performed the experiments. AL contributed with Bt-model of asthma. RA and NC provided the mouse strains, analyzed the results, and contributed to the writing. MR designed the study and wrote the manuscript. All authors read, edited, and approved the manuscript.

## Conflict of Interest

The authors declare that the research was conducted in the absence of any commercial or financial relationships that could be construed as a potential conflict of interest.

## Supplementary Material

FIGURE S1 Effect of liposomal formulation containing allergen plus CpG on antibody production. C57BL/6 WT mice were sensitized s.c. with OVA/Alum on days 0 and 7 and challenged with i.n. OVA on days 14 to establish airway inflammation. Mice were treated subcutaneously with PBS (Allergic); or with encapsulated OVA in DOTAP (OVA/DOTAP) on days 17, 24, and 31 and challenged with i.n. OVA on days 38 and 45. Serum levels of antibodies were determined on day 46. Control group consisted of non-manipulated naive animals. **(A)** Total IgE levels, **(B)** OVA-specific IgE antibodies, **(C)** OVA-specific IgG1 antibodies, and **(D)** OVA-specific IgG2c antibodies. Values represent the mean ± SEM and are representative of two independent experiments. One-way ANOVA: ^∗^*p* < 0.05; ^∗∗^*p* < 0.01.
