# Supplementary figures and images for: Allergen-Specific Immunotherapy With Liposome Containing CpG-ODN in Murine Model of Asthma Relies on MyD88 Signaling in Dendritic Cells
Source: Front Immunol. 2020 Apr 23;11:692. doi: 10.3389/fimmu.2020.00692 (PMC7191058; doi:10.3389/fimmu.2020.00692)

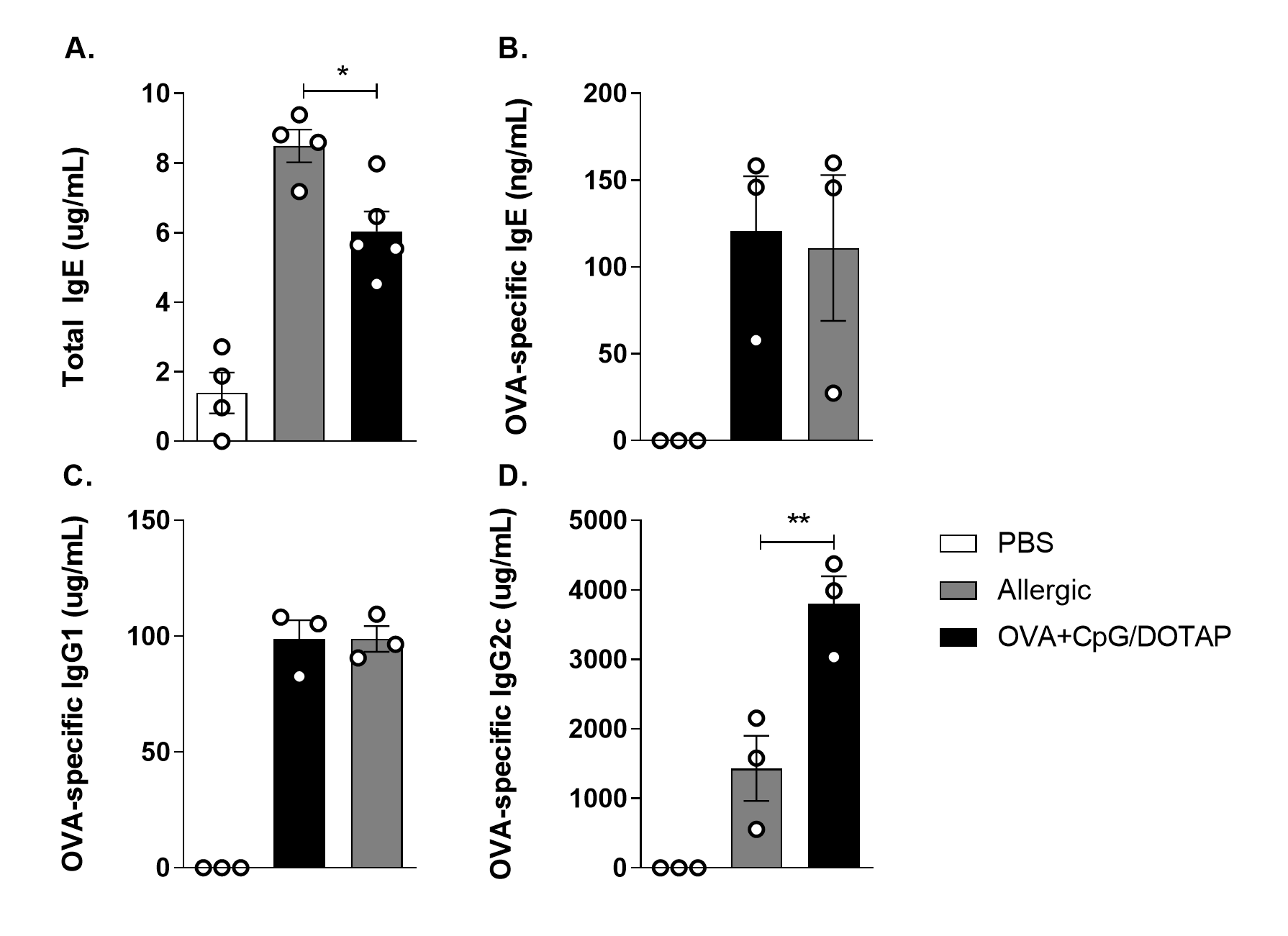

Supplement: FIGURE S1 — Effect of liposomal formulation containing allergen plus CpG on antibody production. C57BL/6 WT mice were sensitized s.c. with OVA/Alum on days 0 and 7 and challenged with i.n. OVA on days 14 to establish airway inflammation. Mice were treated subcutaneously with PBS (Allergic); or with encapsulated OVA in DOTAP (OVA/DOTAP) on days 17, 24, and 31 and challenged with i.n. OVA on days 38 and 45. Serum levels of antibodies were determined on day 46. Control group consisted of non-manipulated naive animals. (A) Total IgE levels, (B) OVA-specific IgE antibodies, (C) OVA-specific IgG1 antibodies, and (D) OVA-specific IgG2c antibodies. Values represent the mean ± SEM and are representative of two independent experiments. One-way ANOVA: ∗p < 0.05; ∗∗p < 0.01. [file Image_1.TIF]
